# Reduced DNA repair in BRCA1 mutation carriers undetectable before onset of breast cancer?

**DOI:** 10.1038/sj.bjc.6603977

**Published:** 2007-09-11

**Authors:** W Vogel, H Surowy

**Affiliations:** 1 Institute of Human Genetics, University of Ulm, Ulm, Germany; 2 Department of Gynecology, University of Ulm, Ulm, Germany

**Sir**,

We read with great interest the article by Kotsopoulos *et al* (2007), which appeared recently in the *British Journal of Cancer*. The authors addressed the question if carriers of BRCA1 mutations can be detected by a reduced capacity to repair DNA double-strand breaks (DSBs) before the onset of the disease. The answer would be important because it could allow to develop such a technique into a predictive test, which could be offered in high-risk families and which might be applicable for a risk assessment for sporadic breast cancer too.

DNA DSBs are repaired by either of the two repair pathways, homologous repair (HR) or nonhomologous end joining (NHEJ). Both processes, when successfully completed, do not leave open ends and, hence, no DNA fragments are left. Mutations in BRCA1 (and BRCA2, which was not studied by Kotsopoulos *et al*, 2007) have been shown to impair HR while NHEJ appears to be largely unaffected (Venkitaraman, 2002). Nonhomologous end joining is available throughout the cell cycle, while the activity of HR appears in early S phase, when the template (the replicated DNA) becomes available.

Kotsopoulos *et al* (2007) used three approaches to interrogate DNA repair capacity and all three did not reveal a difference between BRCA1 mutation carriers and controls. However, we have some concerns about the conclusions and we would like to discuss them in the order they take effect in the cell:

1. The number of H2AX DNA repair granules was studied in lymphocytes prepared directly from the blood sample, which may indeed reflect DNA repair capacity under certain circumstances and may be used to quantitate interindividual variation (Ismail *et al*, 2007). However, the granules detected by antibodies against H2AX are neither specific for DNA DSBs (Bekker-Jensen *et al*, 2006; Au and Henderson, 2007) nor for the HR repair pathway. The assembly of the granules does not depend on the presence of BRCA1 (Foray *et al*, 2003). In consequence, this test can be expected to be very sensitive when early steps of damage recognition are involved but not necessarily for haploinsufficiency of BRCA1. Furthermore, the test has been carried out on lymphocytes in G_0_ in which the amount of BRCA1 is rather low in any case (Venkitaraman, 2001). The use of anti-BRCA1 antibodies might have been more informative, although one may expect to see differences only in cells, which have entered the S phase.
2. The alkaline Comet assay as used by the authors provides a measure predominantly for the frequency of DNA single-strand breaks but also detects DSBs and other DNA damage. When used in the G_0_ phase of the cell cycle to determine DNA repair capacity, the contribution of HR to this capacity is certainly only a very minor point. Although there is evidence that BRCA1 does not function exclusively in HR (Au and Henderson, 2007), one cannot expect to detect small differences in its activity (haploinsufficiency) by the comet assay in G_0_ cells.
3. The third assay used by Kotsopoulos *et al* (2007) was the micronucleus test (MNT) with some variation compared to the most frequently used protocols for the assessment of DNA repair capacity. Among the assays used here, this is probably the only assay, which in principle would be appropriate to detect a difference in HR DNA repair as a consequence of a heterozygous mutation in BRCA1. It has been used with rather consistent results in breast cancer patients with and without family history (Baeyens *et al*, 2004, 2005) and in some studies, a reduced DNA repair capacity could be shown in BRCA1 mutation carriers (Rothfuss *et al*, 2000; Trenz *et al*, 2002).

The differences between the protocol used here and the usual procedure relate to culture time (4 instead of 3 days) and to the timing of the irradiation. Both may not be relevant because similar variations in other assays (Scott *et al*, 1998, 1999; Baeyens *et al*, 2005) did not preclude the detection of a difference between cases and controls. For the timing of irradiation, we can present some systematic data obtained with high-dose rate (2 Gy in 2 min) shifted through the culture time (Figure 1). Much more critical seems the number of binucleated lymphocytes (BNCs), in which the micronuclei were counted. This number was stated to be minimally 200, which is definitely not sufficient to detect a difference of 10% between the two groups, given the variability of micronucleus counts (Vral *et al*, 2002; Patino-Garcia *et al*, 2006). We investigated the influence of the number of BNCs counted on the calculated mean MN frequency by incremental counting of slides from a single blood culture. The MNT was carried out exactly as described by Varga *et al* (2006). Counting was performed by an automated system (Varga *et al*, 2004) to avoid any variation possibly introduced by different observers and to eliminate imperfect reproducibility (coefficient of variation of the machine <5% for counts on the same slide). As obvious from Figure 2, the deviation from the final mean decreases with an increasing number of BNCs and becomes sufficiently small only when more than 1000 BNCs are counted. In earlier studies, a lower limit of 500 BNCs was used and some of the findings were borderline (Baeyens *et al*, 2004; Speit and Trenz, 2004; Patino-Garcia *et al*, 2006). In consequence, counting 200 BNCs, as by Kotsopoulos *et al*, is not sufficient to state a negative result.

By now, ample evidence has accumulated to demonstrate that lymphocytes from breast cancer patients have a slower or reduced repair of DNA DSBs compared to healthy controls. This difference can be detected by analysing chromosome aberrations or micronuclei with various protocols. It can be also seen in breast cancer patients with a BRCA1 mutation. The paper by Kotsopoulos *et al* (2007) states correctly that BRCA1 mutation carriers cannot be detected by the tests they employed using their protocols. They addressed implicitly the important question whether a difference in DNA repair capacity can rather be attributed to the disease status (breast cancer) than to BRCA1 mutation. Investigating BRCA1 mutation carriers without breast cancer should reveal whether the reduced DNA repair capacity arises later on together with the disease or can be detected before its onset and, hence, may represent a direct consequence of the BRCA1 mutation. This second view would be consistent with data from a prospective study, in which increased baseline MN or chromosome aberration frequencies indicated an increased cancer risk (Rossner *et al*, 2005; Bonassi *et al*, 2007).

Two of the tests (H2AX foci and Comet assay) used by Kotsopoulos *et al* (2007) were of questionable relevance concerning the repair of DNA DSBs. The third one, the MNT, was not carried out in a way, which could support a negative result. In consequence, the important question implicated in the study cannot be resolved: whether the reduced DNA repair capacity in breast cancer patients with BRCA1 mutation is due to the cancer or to the mutation. The answer to this question has to await more specifically designed studies.

## Figures and Tables

**Figure 1:**
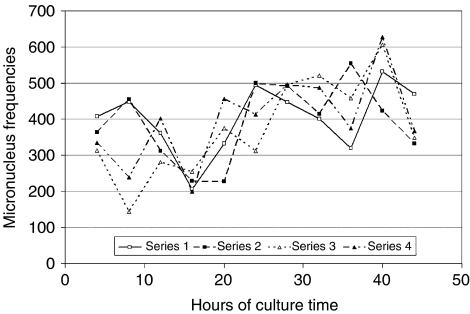
Micronucleus frequencies plotted against time of irradiation. Parallel lymphocyte cultures (72 h) were irradiated with 2 Gy (1 Gy min^−1^) at different time points after setting up the culture. Data points for each proband are connected.

**Figure 2:**
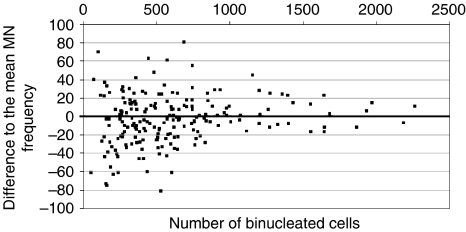
Difference of MN frequency to the final mean MN frequency of that culture plotted against the number of binucleated cells (BNCs) on which the MN frequency was determined. It is obvious that the MN frequencies close in on the final mean at numbers of 1000 or more counted BNCs.
